# What we know about management and organisation of primary dental care in Brazil

**DOI:** 10.1371/journal.pone.0215429

**Published:** 2019-04-18

**Authors:** Tatiana Pereira Santos, Antônio Thomaz Gonzaga Matta Machado, Mauro Henrique Nogueira Guimarães Abreu, Renata Castro Martins

**Affiliations:** 1 Graduate Program in Dentistry, Faculty of Dentistry, Universidade Federal de Minas Gerais, Belo Horizonte, Minas Gerais, Brazil; 2 Department of Preventive and Social Medicine, Faculty of Medicine, Universidade Federal de Minas Gerais, Belo Horizonte, Minas Gerais, Brazil; 3 Department of Community and Preventive Dentistry, Faculty of Dentistry, Universidade Federal de Minas Gerais, Belo Horizonte, Minas Gerais, Brazil; Brown University, UNITED STATES

## Abstract

This cross-sectional study evaluated the management and organisation of primary dental care in Brazil. For this purpose, data from the National Program for Improving Access and Quality of Primary Care (PMAQ-AB) were used. Dentists from 18,114 Oral Health Teams (OHTs) answered a structured questionnaire in 2014. The data were analyzed descriptively and by cluster analysis. Half the Brazilian OHTs (51.0%) undertake planning and programming of activities. The majority of OHTs (66.4%) conducted monitoring and analysis of indicators and oral health information. The majority of OHTs had performed some self-evaluation process in the last 6 months (67.5%) and utilised self-evaluation results for planning and programming actions (71.4%). The OHTs grouped in Cluster 1 demonstrated better management organisation, followed by the teams grouped in Cluster 2. In the Brazilian macro-regions, the more OHTs were grouped in Cluster 1 in the Southeast (87.5%), Northeast (85.4%) and South (82.7%) regions. The majority of OHTs have satisfactory management and organisation. However, some need improvement, mainly in planning and programming actions based on health indicators and self-evaluation. All Brazilian OHTs need to participate in PMAQ-AB and it is important to continue evaluating the data to improve oral health care.

## Introduction

The Brazilian healthcare system, known as the Unified Health System (UHS) (*Sistema Único de Saúde*–SUS, in Portuguese) was implemented through the 1988 Federative Constitution of Brazil. In accordance with Primary Health Care (PHC) and based on the proposals made at the Alma Ata Conference in 1978, the main objective of the UHS is the promotion and universalisation of public health care to the whole population, the realisation of preventive actions and coordination of care based on social context [1,2,3].

To achieve the goals proposed by the UHS, the Family Health Program (FHP), later called the Family Health Strategy (FHS), was introduced in 1994, with the aim of restructuring the PHC, with a focus on comprehensive health care of the population and epidemiological needs of the country, offering the services of health promotion and prevention [4]. The Oral Health Teams (OHTs) were only included in the FHS teams in 2000, restructuring the PHC model. In 2004, the National Oral Health Policy guidelines, called the “Smiling Brazil” Program (*Programa Brasil Sorridente* in Portuguese) was created with actions to reorganise basic health care and the expansion and qualification of specialised care, to improve the oral health problems that had been uncovered in the epidemiological survey *SB Brasil 2003* [5, 6]. After a decade of the “Smiling Brazil” Program, advances in oral health work were observed focusing on educational and permanent education actions; welcoming, bonding and accountability. The main challenges were related to comprehensive health care; extension and improvement of care, teamwork; planning, monitoring, and evaluation of actions, and working conditions. [7].

Critical evaluations of health policies are pertinent to improve the quality of installed health actions and services [8]. It is important to develop research aimed at strengthening primary health care and improving the healthcare system [9]. To strengthen services, it is essential to measure the health system by availability, access, quality and efficiency, and population health by health status, responsiveness, user satisfaction and financial risk protection, through monitoring and evaluating the performance of public healthcare services [10].

In Brazil, the Brazilian Ministry of Health established the National Program for Improving Access to and Quality of Primary Care (Programa Nacional de Melhoria do Acesso e da Qualidade da Atenção Básica–PMAQ-AB, in Portuguese) in 2011. The PMAQ-AB is based on the classic organisation of care and quality proposed by Donabedian, where quality is evaluated using structure, process and results parameters [11]. The PMAQ-AB aims to improve services, both qualitatively and quantitatively, in order to create a quality standard that is comparable at national, regional and local levels [12,13]. The main challenge of the PMAQ-AB is to establish a culture of evaluation among PHC professionals and managers who monitor and evaluate processes and results. OHTs participating in the PMAQ-AB receive financial incentives conditional on performance evaluation of their structure, health indicators, external self-evaluation results and interviews with managers, professionals and users [14].

Monitoring and evaluating actions related the Oral Health Policies is essential to know how the access and quality of services provided, to implement corrective measures and/or improve the actions and services offered in the scope of PHC [14]. So, the aim of this study was to analyse data for the management and organisation of primary oral health care.

## Materials and methods

The study was submitted to and approved by the Human Research Ethics Committee of the Universidade Federal de Minas Gerais, Brazil (protocol number 02396512.8.0000.5149).

This cross-sectional descriptive study used data from the second cycle of PMAQ-AB, conducted by the Brazilian Ministry of Health between 2013 and 2014, and included the entire national territory, covering the macro-regions of Brazil (North, Northeast, Midwest, Southeast and South). This study used secondary data obtained in the external evaluation phase of PMAQ which involved an interview with Brazilian dentists working in OHTs, about the management and organisation of the work process of the OHTs and verification of documents in the PHC units. During the years 2013 and 2014 there were 23,251 OHTs implanted in Brazilian territory. A total of 19,946 OHTs (85.79%) participated in the entire process for certification of the second cycle of PMAQ. At the end of this cycle 1,832 OHTs (9.18%) were disqualified or were unsatisfactory in the program, being excluded of database by the Brazilian Ministry of Health.

A questionnaire used for the interview was created through a Brazilian Ministry of Health partnership with education and research institutions in Brazil. The interviewers were health professionals selected and were trainined to administer the survey across the country. They received a 40-hour training program including survey methods, PHC content and PMAQ questionnaires. After this, all interviewers were submitted to an evaluation to assess their abilities. Each interviewer had one supervisor. An app for mobile devices, which had the questions in electronic format that allowed data collection, was developed by Brazilian Ministry of Health. Data were sent online to the database of the Brazilian Ministry of Health that was responsible for the consistency analysis and certification of OHTs. The interviews with the professionals of the OHTs were voluntary, documents were verified and information previously acquired was inserted into the electronic questionnaires. The questionnaire consisted of mainly dichotomous questions.

The questions were developed according to the principles from primary care [15] that should be incorporated into the OHTs, and included, among other items, questions about the management and organisation processes of dental services in primary care. The questions that were considered are presented at Tables 1 and 2.

**Table 1:** Descriptive analysis of management and organisation of OHTs, according to PMAQ-AB Data, Brazil, 2014.

Variable	Frequency (%)
*Does the OHT perform monthly planning and scheduling activities with a document to prove it*? *(Yes)*	9,238 (51.0)
*Does the OHT conduct monitoring and analysis of indicators and oral health information*? *(Yes)*	12,031 (66.4)
*Does the OHT receive support for the programming and organisation work process*? *(Yes)*	14,303 (79.0)
*Was some self-evaluation process performed by the OHT in the last 6 months*? *(Yes)*	12,218 (67.5)
*Does the OHT receive permanent support from a team or person from the municipal health department*? *(Yes*, *always or since the accession of PMAQ)*	14,925 (82.4)
*Does the OHT program your activities considering*: *the construction of a weekly*, *biweekly or monthly work schedule*? *(Yes)*	16,737 (92.4)
*Does the OHT program your activities considering*: *the goals for primary care according to the municipality*? *(Yes)*	15,541 (85.8)
*Does the OHT program your activities considering*: *data from the primary care information system*? *(Yes)*	14,454 (79.8)
*Does the OHT program your activities considering*: *local information (demand study*, *epidemiological scenario and others)*? *(Yes)*	14,518 (80.1)
*Does the OHT program your activities considering*: *questions related to biological risks and individual*, *family and social vulnerabilities (violence*, *drugs and others)*? *(Yes)*	14,195 (78.4)
*Does the OHT program your activities considering*: *the environmental issues of the territory (including access to land)*? *(Yes)*	11,712 (64.7)
*Does the OHT program your activities considering*: *the challenges mentioned in self-evaluation*? *(Yes)*	12,940 (71.4)
*Is the OHT schedule shared with family health team (FHT) professionals*? *(Yes)*	12,007 (66.3)
*Is the OHT schedule organised to offer oral health education activities in the territory*? *(Yes)*	16,407 (90.6)
*Does the OHT guarantee the schedule of appointments to continue users’ treatment*? *(Yes)*	16,639 (91.9)

**Table 2:** Management organisation of OHTs by cluster, according to PMAQ-AB Data, Brazil, 2014.

Variable	Frequency (%)		
	Cluster 1	Cluster 2	Cluster 3
*Does the OHT perform monthly planning and scheduling activities with a document to prove it*? *(Yes)*	8,599 (56.4)	423 (34.8)	216 (13.1)
*Does the OHT conduct monitoring and analysis of indicators and oral health information*? *(Yes)*	11,574 (75.9)	208 (17.1)	249 (15.1)
*Does the OHT receive support for the programming and organisation work process*? *(Yes)*	13,416 (88.0)	559 (46.0)	328 (19.8)
*Was some self-evaluation process performed by the OHT in the last 6 months*? *(Yes)*	10,879 (71.4)	915 (75.4)	424 (25.6)
*Does the OHT receive permanent support from a team or person from the municipal health department*? *(Yes*, *always or since the accession of PMAQ)*	13,359 (87.6)	872 (71.8)	694 (42.0)
*Does the OHT program your activities considering*: *the construction of a weekly*, *biweekly or monthly work schedule*? *(Yes)*	14,627 (95.9)	1,099 (90.5)	1,011 (61.1)
*Does the OHT program your activities considering*: *the goals for primary care according to the municipality*? *(Yes)*	14,113 (92.6)	864 (71.2)	564 (34.1)
*Does the OHT program your activities considering*: *data from the primary care information system*? *(Yes)*	13,565 (89.0)	556 (45.8)	333 (20.1)
*Does the OHT program your activities considering*: *local information (demand study*, *epidemiological scenario and others)*? *(Yes)*	13,444 (88.2)	830 (68.4)	244 (14.8)
*Does the OHT program your activities considering*: *questions related to biological risks and individual*, *family and social vulnerabilities (violence*, *drugs and others)*? *(Yes)*	12,958 (85.0)	866 (71.3)	371 (22.4)
*Does the OHT program your activities considering*: *the environmental issues of the territory (including access to land)*? *(Yes)*	10,615 (69.6)	847 (69.8)	250 (15.1)
*Does the OHT program your activities considering*: *the challenges mentioned in self-evaluation*? *(Yes)*	11,760 (77.1)	943 (77.7)	237 (14.3)
*Is the OHT schedule shared with family health team (FHT) professionals*? *(Yes)*	10,685 (70.1)	810 (66.7)	512 (31.0)
*Is the OHT schedule organised to offer oral health education activities in the territory*? *(Yes)*	14,280 (93.7)	1,164 (95.9)	963 (58.2)
*Does the OHT guarantee the schedule of appointments to continue users’ treatment*? *(Yes)*	14,316 (93.9)	1,105 (91.0)	1,218 (73.6)

Descriptive statistics were analysed and cluster analysis was performed using the IBM Statistical Package for Social Sciences (SPSS) version 22.0 (IBM SPSS Statistics for Windows, Armonk, NY, USA). Confidence intervals were not calculated because this was a census study. Multivariate agglomerative hierarchy based on the farthest neighbour is an exploratory analysis tool used to organise the data observed in groups (clusters). In this case, analysis of OHTs was based on a combination of independent variables, maximising the similarity of cases within each group (clusters) and maximising the differences between groups [16]. Three types of cluster (including two to four clusters) were formed from the OHTs in Brazil. The three-cluster option was chosen as the best way to interpret the data. After that, the three clusters formed were compared for the Brazilian macro-regions: North, Northeast, Midwest, Southeast and South.

## Results

The final sample was composed by 18,114 OHTs (77.9%).

Table 1 shows the management and organisation of the OHTs associated with planning of evaluation and service to users in the Basic Health Units (BHUs).

Table 2 displays the management organisation of OHTs according to the clusters formed. Overall, the OHTs grouped in Cluster 1 demonstrated the best management organisation, followed by the teams grouped in Cluster 2.

Table 3 shows the distribution of the OHTs in the Brazilian macro regions. The Southeast, Northeast and South regions had more OHTs grouped in Cluster 1.

**Table 3:** Distribution of OHTs in the Brazilian Macro-regions by cluster, according to PMAQ-AB data, Brazil, 2014.

Macro-region	Frequency (%)			
	Cluster 1	Cluster 2	Cluster 3	Total
**North**	930 (73.6)	112 (8.9)	221 (17.5)	1,263 (100.0)
**Northeast**	6,572 (85.4)	572 (7.4)	556 (7.2)	7,700 (100.0)
**Midwest**	1,232 (78.4)	108 (6.9)	232 (14.8)	1,572 (100.0)
**Southeast**	4,401 (87.5)	266 (5.3)	360 (7.2)	5,027 (100.0)
**South**	2,111 (82.7)	156 (6.1)	285 (11.2)	2,552 (100.0)
**Total**	15,246 (84.2)	1,214 (6.7)	1,654 (9.1)	18,114 (100.0)

## Discussion

This study shows the organisation of the work processes of Brazilian OHTs that participated in PMAQ-AB between 2013 and 2014. The majority of the teams organise work processes by planning and programming actions according to community contexts, self-evaluations and team agendas. The majority of OHTs showed good work process performances; however, others must be improved in order to develop an appropriate intervention program.

Half of the Brazilian OHTs in this study reported that they frequently plan and program their activities, and the majority reported that they received permanent support to plan and organise their actions. Almost all of the OHTs declared that they plan their activities based on a work schedule and guarantee that the appointment schedule is maintained to provide continued treatment for users. Planning, implementation and evaluation of actions are important mechanisms to analyse activities in public health. When planning does not occur, activities continue to be part of a curative system. Based on the PHC principle, which represents much more than offer a cure for diseases, the system must be based on both the prevention of disease and the promotion of health, offering the possibility of continued treatment in order to establish full health care [15,17,18].

PHC services must offer health care according to the social-epidemiological profile of the population [19,20,21]. The majority of OHTs reported that they plan their activities considering PHC goals, especially according to data from the primary care information system and local information in the region. Healthcare services must be organised and managed according to a health surveillance system so as to improve local and individual health conditions and to achieve collective results [8,22–24]. The best planning and programming actions are evidence-based, and this evidence comes from epidemiological research, which makes it possible to formulate indicators and parameters to assess the real situation in the community [8,23,24].

The majority of OHTs reported the monitoring and analysis of oral health indicators and information. This monitoring is essential in order to evaluate the performance of healthcare policies and actions, as well as to formulate the criteria necessary to improve health care. Indicators integrated within a continuous process of monitoring and evaluation make it possible to identify emergency situations and implement changes [11,25–28]. Although there have been advances in oral health care since the “Smiling Brazil” Program was introduced, the planning, monitoring and evaluation of actions are far from the everyday routines of the OHT. This remains a challenge that requires mobilisation, engagement and decision-making by both managers and professionals, especially since dental care professionals tend to reproduce the predominant biomedical models. Therefore, further efforts are required in the fields of management, training and permanent education [7].

Another way to improve action planning is to analyse the results of self-evaluations. More than half of the OHTs from the present study reported that they had carried out self-evaluations in the last 6 months and had planned their activities considering the challenges observed in these assessments. Self-evaluation is a crucial element in determining a population’s health needs, identifying target groups and developing new intervention strategies to offer improved oral health services. Thus, self-evaluation should be integrated within the monitoring process [29,30,31].

A little more than half of the OHTs share their schedule with the FHS teams. These results are probably due to the late introduction of the OHTs at FHS, making the integration between the theses teams difficult, since the individual work processes of dentists isolate them inside their offices [7]. It is important that dental professionals in the field of public health work together and offer an integrated healthcare network with greater comprehensive care. The absence of communication between the FHS with the OHTs can compromise the quality and quantity of care [32].

Almost all of the OHTs offer oral health education activities, which focused on how behaviour and changes in lifestyle can impact a population’s overall health, thus leading to the possible best results, according the principles of PHC [15,33].

In the present study, the OHTs in Cluster 1 demonstrated better results than did the OHTs in Cluster 2 and 3 in the management and organisation of primary dental care. Differences in the OHTs’ patterns of distribution in the Brazilian macro-regions were observed, with the Southeastern, Northeastern and Southern regions presenting more OHTs grouped in Cluster 1. Brazil is a developing country and has an extensive territory with high levels of socioeconomic inequality, which explains the different types of management organisation within the Brazilian macro-regions. The Brazilian economy is concentrated in the Southeastern and Southern regions of the country, which provide better incentives for public health. Since the implementation of the “Smiling Brazil” program, the Northeastern region is where the majority of OHTs can be found. These OHTs also receives high incentives from the UHS, which could explain the results of this study [34]. Previous studies, using PMAQ-AB data, also found differences between Brazilian macro-regions when evaluating the relationship between primary and secondary care in public health services [35] and procedures of primary dental health care performed by the OHTs [36]. Both studies observed the best results for OHTs in the Southern and Southeastern regions, reinforcing the great social disparities between the Brazilian regions, even though the UHS does offer actions and services to promote, prevent, treat and improve the health of all Brazilians in all regions [37].

This study showed that the majority of the OHTs have satisfactory management and organisation. However, some need improvements, mainly in the planning and programming of actions, such as the improvement of the population’s health. A limitation of this study is that pay-per-performance models may be biased. The PMAQ-AB invests in the OHTs according to the results obtained by the healthcare teams. Pay-for-performance models can be ineffective, since encouraging well-performing teams will not change their behaviour and the cost of improvement may well prevent poorly performing teams from participating in the programs. However, performance pay systems should be used to drive quality improvement and not merely to reward those who already offer high-performance healthcare services [17,38,39]. Another limitation is that the self-evaluations used dichotomous variables and no specific instructions were provided to the respondents about how to operationalise these questions. Hence, the observations represent a self-perception of whether or not the clinics actually engage in these practices at all. Nonetheless, the sample consisted of dentists working in the OHTs of the UHS, which provides public health care to the entire Brazilian population. It is important to mention that the use of Brazilian public services is associated with a lower family income, a greater number of household residents and more teeth requiring treatment [40]. Therefore, the assessment among PHC professionals and managers who monitor and evaluate processes and results is essential to the production of measures that assist in decision-making and improvements in services. This study is important and innovative, because it shows how management and organisation in primary dental care is working in Brazil and how new polices can be traced in order to improve the quality of services and, consequently, oral health care.

## Supporting information

S1 File Dataset. BancoTatiana_Plos One.
(SAV)
